# *miR-618* rs2682818 C>A polymorphism decreases Hirschsprung disease risk in Chinese children

**DOI:** 10.1042/BSR20193989

**Published:** 2020-05-11

**Authors:** Yi Zheng, Tongyi Lu, Xiaoli Xie, Qiuming He, Lifeng Lu, Wei Zhong

**Affiliations:** Department of Pediatric Surgery, Guangzhou Institute of Pediatrics, Guangdong Provincial Key Laboratory of Research in Structural Birth Defect Disease, Guangzhou Women and Children’s Medical Center, Guangzhou Medical University, Guangzhou 510623, Guangdong, China

**Keywords:** Hirschsprung disease, miR-618, polymorphism, susceptibility

## Abstract

MicroRNAs (miRNAs) are endogenous non-coding small RNAs that play an important role in the development of many malignant tumors. In addition, recent studies have reported that single nucleotide polymorphisms (SNPs) located in the miRNA functional region was inextricably linked to tumor susceptibility. In the present study, we investigated the susceptibility between *miR-618* rs2682818 C>A and Hirschsprung disease (HSCR) in the Southern Chinese population (1470 patients and 1473 controls). Odds ratios (ORs) and 95% confidence intervals (CIs) were used for estimating the strength of interrelation between them. We found that the CA/AA genotypes of *miR-618* rs2682818 were associated with a decreased risk of HSCR when compared with the CC genotype (OR = 0.84, 95% CI = 0.72–0.99, *P*=0.032). Based on the stratified analysis of HSCR subtypes, the rs2682818 CA/AA genotypes were able to significantly lessen the risk of HSCR compared with CC genotype in patients with long-segment HSCR (adjusted OR = 0.70, 95% CI = 0.52–0.93, *P*=0.013). In conclusion, our results indicated that the *miR-618* rs2682818 C>A polymorphism was associated with a reduced risk of HSCR in Chinese children, especially in patients with long-segment HSCR (L-HSCR) subtype.

## Introduction

Hirschsprung disease (HSCR), also known as aganglionosis, is a common congenital digestive tract disease in pediatric surgery [1]. It is a significant racial difference in the incidence of HSCR [2]. The incidence in Asians is approximately 2.8/10000, while the incidence of Hispanics is approximately 1/10000 [3–5]. In addition, the incidence of HSCR is also significantly related to gender, with a male to female ratio of 4:1 [6]. Clinically, according to the length of the ganglion segment, HSCR can be divided into three types, including short-segment HSCR (S-HSCR), long-segment HSCR (L-HSCR) and total colon aganglionosis (TCA) [7,8]. The overwhelming majority of HSCRs exhibit sporadic traits, and approximately 20% of HSCR cases present a familial genetic pattern [7,9,10]. HSCR is characterized by siblings whose risk of recurrence is 3–17%, with different incidence rates being significantly associated with gender, ganglion segment length and familial [10,11]. In recent years, more than a dozen genes related to the pathogenesis of HSCR were confirmed, including *RET* [12], *EDNRB* [13], *SOX10* [14], *GDNF* [15], *EDN3* [16], *PHOX2B* [17,18], etc. However, these gene mutations associated with the onset of HSCR are only half of current HSCR cases [6]. Therefore, the genetic model of HSCR is a multifactorial genetic disease, and its specific pathogenesis remains to be further explored [19,20].

MicroRNAs (miRNAs) are single-stranded, non-coding RNAs containing 17–25 nucleotides that specifically bind to the 3′ untranslated region (UTR) region of mRNA and regulate the related gene expression [21,22]. It is estimated that approximately 30% of genes in the human genome regulated by miRNAs [23,24]. Single nucleotide polymorphisms (SNPs) are the most common, numerous and widely distributed variants of the human genome [25]. By interfering transcription, processing of pri-miRNA and pre-miRNA, SNPs in the functional region can affect the function of miRNA, thereby regulating the expression of downstream genes [26].

Recent studies have shown that *miR-618* deregulation is closely related to tumorigenesis, including hepatocellular carcinoma [27], thyroid cancer [28], breast cancer [29], bladder cancer [30] and Barrett’s esophageal cancer. The SNP rs2682818 located on the stem-loop sequence of the *miR-618* precursor, so it can affect and/or alter the secondary structure of *pre-miR-618*, which in turn affects the *miR-618* expression, thereby affecting the association of *miR-618* with cancer risk [31,32]. In recent years, more and more reports have illustrated that SNP rs2682818 is closely related to the susceptibility of a variety of diseases, including follicular lymphoma [31], acute lymphocytic leukemia [33], colorectal cancer [34] and breast cancer [32]. However, the correlation between SNP rs2682818 and HSCR risk is still unclear. Therefore, we performed this case–control study with 1470 cases of HSCR and 1473 control subjects to assess the association between *miR-618* rs2682818 C>A polymorphism and HSCR susceptibility in Chinese children.

## Materials and methods

### Study subjects

In the present study, 1470 HSCR cases were collected from 2010 to 2015 and 1473 controls. These patients were diagnosed as HSCR in the pediatric outpatient clinic of Guangzhou Women and Children Children’s Hospital (Guangzhou, China). The description of specific diagnostic criteria for HSCR could be found in our previous studies [35]. Participants without a history of HSCR and neurological disease were used as controls. According to the segment length of the ganglion, the case groups were divided into three subgroups, including S-HSCR, L-HSCR and TCA. The present study was approved by the Guangzhou Women and Children Children’s Hospital Institutional Review Board. Written informed consent was obtained from the guardian of all participants. The ethics certificate number is: 201943800.

### SNP genotyping

For the selection of potential functional polymorphisms, we have described in detail in our previous study [36]. Genomic DNA was extracted from venous blood and paraffins samples using the TIANamp Blood Genomic DNA Kit and TIANquick FFPE DNA Kit (TIANGEN Biotech Co. Ltd., Beijing, China) [37]. Subsequently, we amplified the genomic DNA samples by ABI-7900 real-time quantitative PCR instrument (Applied Biosystem, Foster City, CA) and performed TaqMan genotyping on the selected polymorphisms [38–40]. We randomly selected 10% of the DNA samples for the second genotyping. The consistency of all replicate samples was 100% to ensure the accuracy of the data [41].

### Statistical analysis

Statistical analysis in the present study was performed by SAS 9.4 software (SAS Institute Inc, Cary, NC, U.S.A.). A two-sided chi-square test was used to verify the distribution of samples characteristics between all cases and controls group. The goodness-of-fit chi-squared test was used to examine that the genotype frequencies in the controls were consistent with the Hardy–Weinberg equilibrium (HWE). By multivariate logistic regression analysis, the adjusted odds ratio (OR) and 95% confidence interval (CI) were calculated and then used for assessing the association between *miR-618* rs2682818 C>A polymorphism and HSCR risk. Only when *P*<0.05 could be considered statistically significant.

## Results

### Association between *miR-618* rs2682818 polymorphism and HSCR susceptibility

In the present study, 1330 cases and 1453 controls were successfully genotyped. The frequency distribution of rs2682818 C>A genotype in the control groups was consistent with HWE (*P*=0.793). The case group and the control genotype frequencies in the present study were shown in Table 1. We found that the *miR-618* rs2682818 C>A polymorphism was associated with a reduced risk of HSCR (OR = 0.84, 95% CI = 0.72–0.99, *P*=0.032). In addition, *miR-618* rs2682818 C>A polymorphism showed significant correlations with HSCR risk in the additive (adjusted OR = 0.88, 95% CI = 0.77–0.99, *P*=0.038) and dominant models (adjusted OR = 0.85, 95% CI = 0.72–0.99, *P*=0.036).

**Table 1:** Association between *miR-618* rs2682818 C>A polymorphism and HSCR susceptibility

Genotype	Cases (*n*=1330)	Controls (*n*=1453)	*P*^1^	Crude OR (95% CI)	*P*	Adjusted OR (95% CI)^2^	*P*^2^
rs2682818C>A (HWE = 0.793)							
CC	788 (59.25)	798 (54.29)		1.00		1.00	
CA	461 (34.66)	555 (38.20)		**0.84 (0.72–0.99)**	**0.032**	0.85 (0.72–1.01)	0.058
AA	81 (6.09)	100 (6.88)		0.82 (0.60–1.12)	0.209	0.81 (0.58–1.11)	0.184
Additive			0.029	**0.87 (0.77–0.99)**	**0.029**	**0.88 (0.77–0.99)**	**0.038**
Dominant	542 (40.75)	655 (45.08)	0.021	**0.84 (0.72–0.97)**	**0.021**	**0.85 (0.72–0.99)**	**0.036**
Recessive	1249 (93.91)	1353 (93.12)	0.397	0.88 (0.65–1.19)	0.399	0.86 (0.63–1.17)	0.332

1 χ^2^ test for genotype distributions between HSCR patients and controls.

2 Adjusted for age and gender. For values in bold, *P*< 0.05.

### Subtype analysis of the relationship between rs2682818 polymorphism and HSCR risk

The different subclinical features of HSCR are classified according to the length of the ganglion. Compared with the rs2682818 CC genotype, the CA/AA genotypes were able to remarkably reduce the risk of HSCR in patients with L-HSCR (adjusted OR = 0.70, 95% CI = 0.52–0.93, *P*=0.013). However, no significant correlation was found in patients with S-HSCR and TCA (Table 2).

**Table 2:** Stratification analysis for the association between *miR-618* rs2682818C>A and HSCR susceptibility (by subtype)

Variables	rs2682818 (cases/controls)		Crude OR (95% CI)	*P*	Adjusted OR^1^ (95% CI)	*P*^1^
	CC	CA/AA				
S-HSCR	545/798	408/655	0.91 (0.77–1.08)	0.274	0.93 (0.78–1.10)	0.385
L-HSCR	162/798	90/655	**0.68 (0.51–0.89)**	**0.006**	**0.70 (0.52–0.93)**	**0.013**
TCA	42/798	24/655	0.70 (0.42–1.16)	0.166	0.72 (0.42–1.21)	0.212

1 Adjusted for age and gender with omitting the corresponding stratification factor. For values in bold, *P*< 0.05.

## Discussion

*miR-618* is a small molecule consisting of 98 nucleotides located on chromosome 12. In recent years, more and more reports have reported that *miR-618* is closely related to various malignant tumors. Song et al. [42] found that *miR-618* was down-regulated in metastatic androgen-independent prostate cancer, and patients with low *miR-618* had a poor prognosis. It was worth mentioning that overexpression of *miR-618* could induce MET (mesenchymal-to-epithelial transition) by binding to *FOXP2* (*Forkhead box p2*), and inhibiting the migration and invasion of prostate cells. A study from Shi et al. [43] reported that *miR-618* was down-regulated in gastric cancer tissue, whereas up-regulation of *miR-618* expression inhibited migration and invasion of gastric cancer cells. These studies suggest that the low expression of *miR-618* may be negatively correlated with the ability of malignant tumors to metastasize.

The SNP rs2682818 is a part of the *miR-618* precursor stem loop sequence, so the rs2682818 polymorphism can alter the secondary stem-loop structure of *miR-618*, thereby affecting *pre-miR-618* processing and expression levels [31,44]. It is worth mentioning that Zhu et al. have reported that *miR-146a* rs2910164 homozygous GG expression level is higher than CC, which indicates that C-to-G in rs2910164 may change the precursor of *miR-146a*, thereby improving mature *miR-146a* expression level in HSCR [45]. They suspected that the amount of mature miRNA changed by somatic mutation could affect HSCR risk through by inhibiting *TRAF6* and *IRAK1*, key adapter molecules downstream of the Toll-like and cytokine receptors, which were frequently rearranged with *RET* proto-oncogene [46]. This is very similar to the expression pattern of *miR-618*, and it is worth further researching the role of rs2682818 in HSCR. Fu et al. [31] reported that the rs2682818 variant A allele was associated with decreased expression of mature *miR-618* and increased susceptibility to follicular lymphoma. Interestingly, the rs2682818 C>A polymorphism presented a protective effect in chronic lymphocytic leukemia [47]. Chen et al. found that people with AA or AC genotype had a lower risk of colorectal cancer than CC genotype [34]. In addition, a recent meta-analysis study by Feng et al. [47] showed that rs2682818 of *miR-618* was associated with breast cancer and *miR-618* could be a potential biomarker. These studies indicate that rs2682818 variant A allele can influence the *miR-618* expression, thereby affecting the risk of susceptibility to some malignancies or other diseases. The *RET* gene was the first gene discovered to be recognized as a major player in the pathogenesis of HSCR [48]. A meta-analysis study from Liang et al. reported that the SNPs of (rs1800858, rs1800860, rs10900297 and rs2435357) *RET* polymorphisms was significantly associated with an increased risk of HSCR [49]. Subsequently, Zhang et al. [35] found that the risk of *RET* SNPs in S-HSCR patients was significantly higher than in patients with L-HSCR and TCA in the sporadic cases. However, we found that the rs2682818 polymorphism significantly reduced HSCR susceptibility in the L-HSCR population in the stratified analysis based on ganglion length. In addition, early research found that patients with long segments were characterized by autosomal dominant inheritance, and patients with short segments were characterized by recessive or multifactorial inheritance [50]. Our results demonstrated that *miR-618* rs2682818 C>A significantly reduced the risk of HSCR susceptibility in both dominant and additional models. This suggests that *miR-618* rs2682818 C>A may be a dominant inheritance of non-*RET* gene variants.

To the best of our knowledge, this was the first study to explore the association of rs2682818 polymorphism in *miR-618* with HSCR susceptibility. In the present study, the proportion of males in the case group was 83.67%, and that in the control group was 34.35%. After adjusting for age and gender, the polymorphism of rs2682818 CA genotype was no significant association related to HSCR risk comparing with CC genotype. However, the *P*-value is bounded by a critical value and has a certain statistical significance. This indicates that participants with different genders of CA genotype have a close relationship with susceptibility to HSCR. On the other hand, this also implies that the small sample sizes, all of them from the southern Han population, which will bring a certain deviation to judge the association of rs2682818 genotype and HSCR susceptibility risk.

In addition, some limitations need to be mentioned in this experiment. First, we have not yet analyzed the association of HSCR with environmental factors, including the effects of diet, geographic location, etc. Second, there is still a lack of in-depth exploration of *miR-618* rs2682818 and HSCR sensitivity mechanisms, which has potential implications for HSCR therapy.

In summary, in the present study, we found that the *miR-618* rs2682818 C>A polymorphism could reduce the risk of HSCR susceptibility, especially in patients with L-HSCR. Next, multiregional and multicenter collaboration is needed to expand the sample sizes and verify this susceptibility, thus providing a new direction for clinical research.

## Abbreviations

CI: confidence interval
HSCR: Hirschsprung disease
HWE: Hardy–Weinberg equilibrium
L-HSCR: long-segment HSCR
miRNA: microRNA
OR: odds ratio
S-HSCR: short-segment HSCR
SNP: single nucleotide polymorphism
TCA: total colon aganglionosis
UTR: untranslated region
